# A Simple Biochemical Method for the Detection of Proteins as Biomarkers of Life on Martian Soil Simulants and the Impact of UV Radiation

**DOI:** 10.3390/life13051150

**Published:** 2023-05-09

**Authors:** Yongda Li, David A. Collins, Konstantinos Grintzalis

**Affiliations:** School of Biotechnology, Dublin City University, D09 Y5NO Dublin, Ireland

**Keywords:** protein assay, Bradford, Martian soil simulant, extraction, UV radiation, life signatures

## Abstract

The search for life on other planets relies on the detection of biosignatures of life. Many macromolecules have been suggested as potential targets, among which are proteins that are considered vital components of life due to their essential roles in forming cellular structures, facilitating cellular communication and signaling, and catalyzing metabolic reactions. In this context, accurate quantification of protein signatures in soil would be advantageous, and while several proposed methods exist, which are limited by their sensitivity and specificity, their applicability needs further testing and validation. To this aim, we optimized a Bradford-based assay with high sensitivity and reproducibility and a simple protocol to quantify protein extracted from a Martian soil simulant. Methods for protein spiking, extraction, and recovery were optimized, using protein standards and bacterial proteins as representative models. The proposed method achieved high sensitivity and reproducibility. Taking into account that life remains could exist on the surface of Mars, which is subjected to UV radiation, a simulation of UV exposure was performed on a spiked soil simulant. UV radiation degraded the protein spike, thus highlighting the importance of searching for the remaining signal from degraded proteins. Finally, the applicability of the method was explored in relation to the storage of the reagent which was stable even up to 12 months, thus making its application possible for future planetary exploration missions.

## 1. Introduction

Planetary exploration focuses on the search for traces of life as signatures to investigate habitability on other planets. Macromolecules, such as lipids [1], nucleic acids [2], and proteins and amino acids [3], could be considered as life signatures for exploring the existence of life on other planets. Even though extra-terrestrial life may not be similar in chemical structure to life on Earth, it is still essential to use macromolecules which are based on life as we know as references for searching potential biosignatures on soils from other planets. Proteins are a class of complex biomolecules that are essential to life as we know it. They play critical roles in cellular processes such as catalysis, metabolism, and signaling. Due to their diverse functional roles and intricate structural complexity, proteins are considered ideal biomarkers for the detection of extraterrestrial life [4]. Notably, proteins constitute the most abundant biomolecule in terms of mass, accounting for approximately 55% of the dry weight in bacterial species such as *E. coli* [5]. Thus, the detection and characterization of proteins in extraterrestrial environments could provide a reliable indication of biological activity beyond Earth. Proteins are universal in nature, with many shared characteristics across all living organisms. As such, they offer a common framework for the search for life in a wide variety of environments. In this context, proteins could be an excellent candidate category of macromolecules for the detection of life signatures on potential areas on the soil surface of planets under investigation as habitable regions.

There are several methods to quantify proteins: all account for different reaction mechanisms and, thus, susceptibilities to interference. The most commonly used methods to quantify proteins are the Kjeldahl method, the Biuret method (i.e., the BCA assay and the Lowry assay), the colorimetric (dye-based) methods (i.e., the Bradford assay) and the fluorescent dye methods (i.e., the EZQ fluorescent assay and the qubit protein assay) [6].

Among these methods, the Kjeldahl method is a reliable and well-established technique to measure total nitrogen content in a wide range of samples which is relatively simple and straightforward, with a low cost of equipment and reagents [7]. However, the Kjeldahl method requires the use of concentrated sulfuric acid, which may not be practical or safe to use in the harsh Martian environment. The method also involves several steps, including digestion and titration, which may be difficult to perform in a low-gravity or high-radiation environment [7]. The Lowry method is based on the reaction of copper ions resulting from the oxidation of peptide bonds with the Folin–Ciocalteu reagent [8]. Such an approach is very prone to copper ion interference from the soil matrix. Additionally, the quantification of organic N in soil samples could be an approach to expressing protein content; however, the common methods require a significant amount of incubation time and are also prone to inorganic nitrogen (i.e., nitrate and ammonium) [9]. The fluorescent dye methods are using special fluorescent dyes to combine with proteins and directly detect the increase in fluorescence associated with the bound dye. These assays have excellent sensitivity and can be adapted for automated handling in high-throughput applications [10]. However, they require specialized equipment, which is a big challenge in developing a simple assay. Alternatively, the highly cited Bradford method relies on a more specific reaction of the Coommassie Brilliant Blue (CBB) reagent with amino acids of proteins [11]. We have previously explored the mechanism of the Bradford assay [12] and recently developed a simplified approach [13].

The flexibility and accuracy of the Bradford assay make it a widely-used tool with diverse applications in various fields such as geosciences, agriculture, biotechnology, and medical research. In geosciences, the Bradford assay can be used to quantify the protein content of the soil, a critical factor in understanding nutrient cycling and biological activity in various soil types [14]. This information is valuable in improving soil management and agricultural practices. Similarly, in agriculture, the Bradford assay can be utilized to monitor the quality and quantity of protein in animal feed, an important aspect of ensuring animal growth and health [15]. It can also be used to determine the protein content of plant-based foods, which is essential for human nutrition [16]. In the context of astrobiology, it holds the potential to detect and quantify proteins in extra-terrestrial environments, opening new possibilities for exploring the potential for life beyond Earth.

Designing novel methods for targeted assays in the context of planetary exploration is a significant advancement of the field, which aims to save significant resources and generate conclusive results and findings. The complexity of proteins results in the richness of information that can be extracted from the soil, but there are not many techniques for analyzing this information [17]. Regarding this situation, soil proteomics was introduced at the molecular level for analyzing proteins in soil. Generally, the methods for soil proteomics include extraction and analytical methods, and the aim of the analytical techniques is to determine the selection of the appropriate extraction method [17]. For example, if the aim of the analytical method is the evaluation of the function of proteins, including enzyme activity, then the extraction method aims to protect the function of the proteins as much as possible. Conversely, if the aim of the analytical method is the comparison of the molecular size or the immunological detection, then the extraction method could be less conservative [18]. Among the analytical methods, polyacrylamide gel electrophoresis (PAGE) is considered an economical and straightforward approach with high sensitivity to detect proteins. It also provides compatibility with further analysis, such as mass spectrometry [19]. In this method, 2,2,2-trichloroethanol (TCE) can provide the possibility of the visible fluorescent detection of proteins in polyacrylamide gels [20]. It relies on the reaction of tryptophan in proteins with trihalocompounds to produce fluorescence, allowing for the detection of protein bands without the need for staining. Furthermore, the use of TCE allows for the assessment of integral membrane proteins which cannot be stained with the commonly used CBB reagent [20,21]. Another proteomics that can be used for analyzing soil protein is metaproteomics. It can capture the full range of proteins present in a given environment over a specific time period [22]. However, complex soil conditions, such as humic interference, can affect the result of the analysis. Additionally, transcriptomics, which involves the analysis of RNA transcripts, could also be used to infer the presence and activity of proteins in soil [23]. By identifying transcripts that code for specific proteins, transcriptomics can provide insight into the genes that are actively expressed in the soil environment. However, it is important to note that the use of transcriptomics to infer protein expression may be limited by factors such as post-transcriptional modifications, alternative splicing, and protein stability [24].

The primary objective of this study was to optimize a simple and cost-effective Bradford-based assay for the accurate quantification of proteins extracted from Martian soil simulant, with the ultimate aim of assessing the applicability of the method for future planetary exploration missions. The successful application of this method could significantly improve our ability to detect potential biosignatures on Mars and other planets, thereby advancing our understanding of the origin and evolution of life in the universe. As expected on the Martian soil’s surface, UV radiation was explored as a harsh condition for protein signatures [25]. Our hypothesis was that the optimized assay would demonstrate high sensitivity and reproducibility, enabling the detection of protein signatures that could be preserved on the Martian surface, despite the harsh effects of UV radiation exposure. Overall, this study contributes to the development of new technologies for the search for extraterrestrial life, which is a fundamental scientific question with broad implications for our understanding of the universe and our place in it.

## 2. Materials and Methods

The Bradford-based assay was used to quantify the amount of protein extracted from a Martian soil simulant. The assay was optimized using protein standards and bacterial proteins as more representative samples. The optimization process involved spiking, extraction, and recovery steps to ensure the accuracy and reproducibility of the results. The spiked soil samples were subjected to a range of protein concentrations, and the assay’s sensitivity was evaluated. The optimized assay was then used to quantify the amount of protein in Martian soil simulant samples.

### 2.1. Protein Quantification Assay

The method is based on the electrostatic reaction of proteins with the Coomassie Brilliant Blue G (CBB) reagent under acidic conditions [13]. CBB was prepared in 2 M HCl (60 mg CBB per 100 mL 2 M HCl, filtered and kept light-protected). The reagent was diluted with equal volume with 2 M HCl immediately prior to use (designated as CBB:2M HCl). In total, 200 μL from a protein standard or an appropriately diluted sample (extract from soil) were mixed with 50 μL CBB:2 M HCl reagent and after ten minutes of incubation, the absorbance was measured at 610 nm using a microplate reader. As a reagent blank, 200 μL ddH_2_O was used instead of the sample/protein standard. The mixtures were incubated for 10 min at room temperature and the absorbance was measured at 610 nm. The net absorbance (derived from the absorbance difference of the sample minus the reagent blank) was converted to protein concentration equivalents using the corresponding standard curve.

### 2.2. Soil Simulant and Spiking

**Figure 1:**
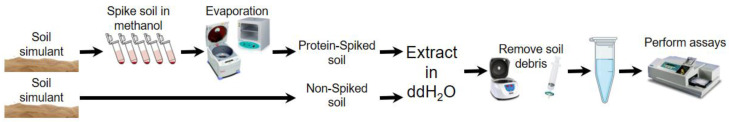
Spiking, extraction, and quantification steps for recovery of protein from soil simulants.

The JSC Mars-1A Martian Regolith Simulant was kindly provided by NASA’s Johnson Space Centre and used as the soil matrix spiked in this study (Figure 1). It is a Martian regolith simulant developed by NASA’s Johnson Space Center to support scientific research, engineering studies, and education. It consists of the <1 mm fraction of palagonitic tephra, a glassy volcanic ash altered at low temperatures from Pu’u Nene, a cinder cone on the Island of Hawaii. The studies of the cone indicate that the tephra is a close spectral analog to the bright regions of Mars, making JSC Mars-1A a useful tool for simulating Martian soil in laboratory experiments [26].

To assess the capability of the JSC Mars-1A simulant to support the detection of proteins, two different conditions were applied: protein-spiked and unspiked. In the spiked condition, a known amount of protein solution (50 μL of different concentrations) was dissolved in methanol and mixed with 50 mg of JSC Mars-1A. The resulting mixture was then subjected to a drying process using either a speed vacuum concentrator or an oven (Figure 1). The extraction of the spiked protein was carried out by adding 1 mL ddH_2_O into the dried mixture and then vortexing vigorously, and the soil debris was subsequently removed by either centrifugation or filtration (0.2 μm). The unspiked condition, which served as a negative control, followed the same procedure but without the addition of any protein solution.

### 2.3. Bacteria Culture and Preparation

Luria Bertani (LB) broth was prepared by dissolving 20 g of LB in 1 liter of ddH_2_O. The broth was transferred to Duran bottles and sterilized by autoclaving for 15 min at 121 °C. Sterile LB broth media was stored at 4 °C until use to prevent any contamination. *E. coli* was cultured at 30 °C under agitation at 200 rpm. From a liquid culture in LB broth media, a bacterial suspension was prepared in Eppendorf tubes and centrifuged (to remove media) at 6000× *g* for 5 min at room temperature. The media supernatant was removed, and the pelleted bacteria cells were resuspended in ddH_2_O. Bacteria were precipitated again by centrifugation at 6000× *g* for 5 min and the supernatant was discarded. For generating a protein homogenate, the pelleted cells were re-suspended in 100 mM NaOH and homogenized by heating at 95 °C in a water bath for 2 h. The protein homogenate was cleared from cellular debris by centrifugation at 6000× *g* at room temperature for 5 min and the clear protein supernatant was collected and assayed for protein and used for spiking.

### 2.4. Materials

All protein standards used in this study were of the highest purity and quality and purchased from Sigma-Aldrich (St. Louis, MO, USA): bovine serum albumin (catalog number: 15561020); transferrin (catalog number: t13342); glucose oxidase (catalog number: g2133); fetuin (catalog number: 10344026); invertase (catalog number: 2326157); anza alkaline phosphatase (catalog number: ivgn2204). Brilliant Blue G 250 (catalog number: 20279) was purchased from Sigma-Aldrich (St. Louis, MO, USA), and hydrochloric acid (12 M HCl, diluted to 2M in ddH_2_O) was purchased from Fisher Chemical (Pittsburgh, PA, USA). Luria Bertani base powder (catalog number: 12795084) was purchased from Sigma-Aldrich (St. Louis, MO, USA).

## 3. Results

**Figure 2:**
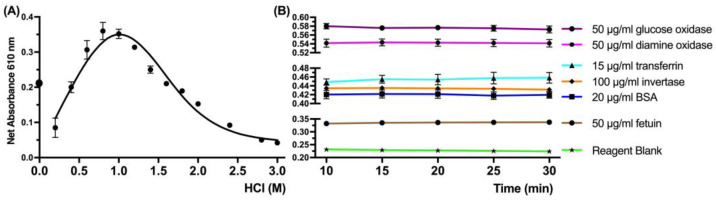
Optimization of the protein assay. (**A**) The impact of acidity on the reaction was observed with BSA (20 μg BSA/mL) mixed with 25 μL CBB and different concentrations of HCl (M). (**B**) The stability of the signal over time was observed for the chosen acidity concentration for different proteins. Data represent average ± SD (N = 3).

Initially, the acidity of the CBB assay reaction was determined from 0 to 3 M HCl (Figure 2A). For the Bradford assay, the concentration of protons is crucial and a sufficient concentration is required for the reaction; however, extensive acidity resulted in interference and a decrease in the sensitivity of the method. Additionally, the time of incubation of the reaction was explored with a series of protein standards, and the reaction signal remained stable from five up to at least thirty minutes, which allowed a significant time window for the feasibility of the method and a fast test (Figure 2B).

**Figure 3:**
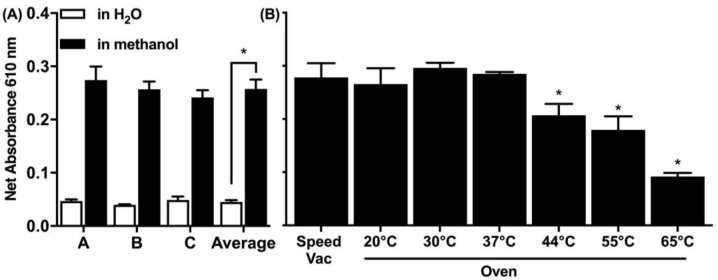
Optimization of the spiking in relevance to (**A**) the solvent and (**B**) the drying method. A. BSA spiked to the soil simulant was spiked in ddH_2_O or 90% methanol at 1 μg BSA/mg soil and was recovered following extraction in 1 mL ddH_2_O. Data represent average ± SD (N = 3) for three independent experiments (A, B, C) and their average and were considered statistically significant (*) by Student’s *t*-test compared between extraction solvents. (**B**) BSA was spiked in 90% methanol (at 1 μg BSA/mg soil) under different drying options and was recovered following extraction in 1 mL ddH_2_O. Data represent average ± SD (N = 3) and were considered statistically significant by Student’s *t*-test compared to the speed vac drying method.

In relevance to the spiking of the soil matrix, two spiking solvents were explored: water and 90% methanol (Figure 3A). Methanol is a volatile solvent and evaporates faster and results in higher spiking efficiency, which was more effective and the preferred spiking approach for all consequent experiments. There was a five-fold increase in the signal detected when the protein was spiked in methanol compared to spiking in water. Furthermore, two drying methods were explored in a range of temperatures using an oven or a rotary vacuum concentrator (speed vac) (Figure 3B). The speed vac operates at a set room temperature while drying in the oven and was tested up to 65 °C. Both the speed vac and the oven at drying temperatures below 37 °C were equally effective.

**Figure 4:**
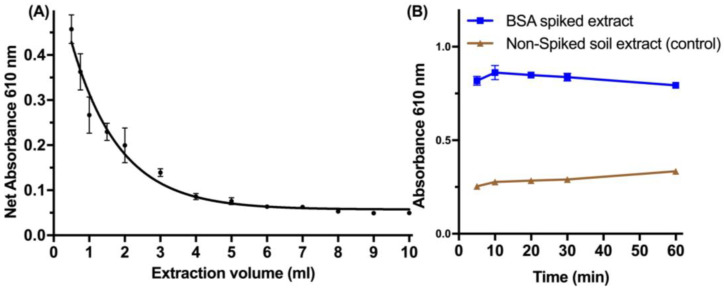
Optimization of the extraction step. (**A**) The volume (mL) of 90% methanol was tested for the extraction of spiked BSA (1 μg BSA/mg soil). (**B**) The time of extraction for 1 mL 90% methanol was explored for the recovery of the spiked BSA (1 μg BSA/mg soil). Data represent average ± SD (N = 3).

Following the optimization of the spiking of protein, the extraction efficiency was investigated regarding the volume and time of extraction (Figure 4). Small volumes (<1 mL) of ddH_2_O are equally effective for the reproducible extraction of spiked protein from the soil, which allowed the miniaturization of the developed method (Figure 4A). The time of extraction during vortexing was effective to as low as 10 min for complete recovery of the protein (Figure 4B).

**Figure 5:**
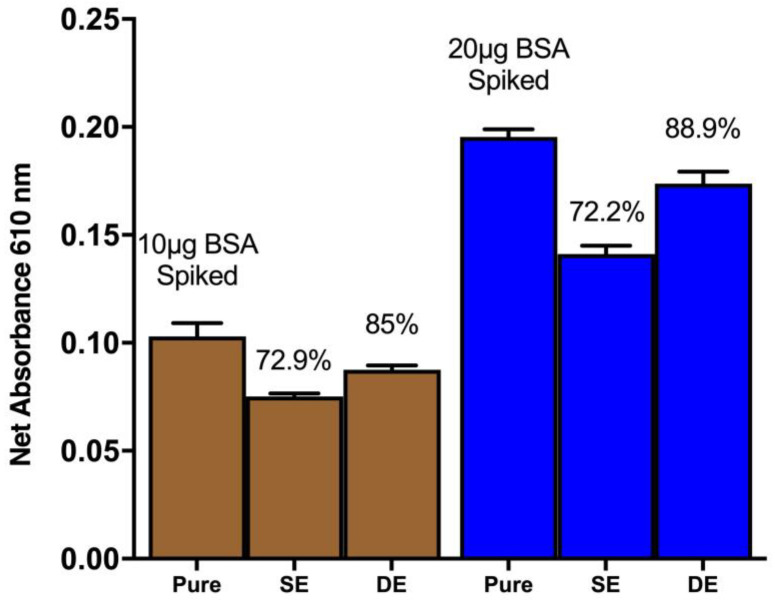
The recovery of protein from the spiked soil simulants (Pure: Pure BSA in ddH_2_O; SE: single extraction; DE: double extraction). Data represent average ± SD (N = 3), and BSA was spiked at 10 μg and 20 μg.

The recovery of proteins from spiked soil simulants was investigated through single and double extraction methods (Figure 5). The results revealed that a single extraction method can yield up to 72% recovery of the spiked proteins from the soil simulants. However, a double extraction method, involving the collection of supernatant after the first vortex and centrifugation step followed by another round of extraction with ddH_2_O, resulted in an 89% recovery of the spiked signal.

**Figure 6:**
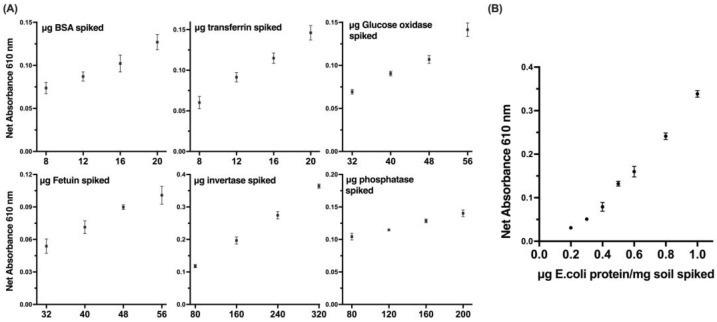
Linearity of the protein assay for a series of protein standards (**A**) and bacterial protein (**B**) spiked on the soil simulant. Data represent average ± SD (N = 3).

The sensitivity of a developed method for detecting proteins was evaluated by using purified protein standards, such as bovine serum albumin (BSA) and fetuin, which are commonly used as protein standards in various biochemical assays (Figure 6A). These standards were used to establish a calibration curve and determine the limit of detection (LOD) of our method. In addition to the purified protein standards, the utilization of bacterial protein in the sensitivity evaluation of our method provided a more representative scenario for the detection of microbial protein traces (Figure 6B). The bacterial protein used in our study was extracted from an *E.coli* culture of a known concentration and was used to evaluate the sensitivity and specificity of our method in detecting microbial proteins. The results of the study showed that the developed method displayed significant linearity, reproducibility, and a remarkably low level of detection.

**Figure 7:**
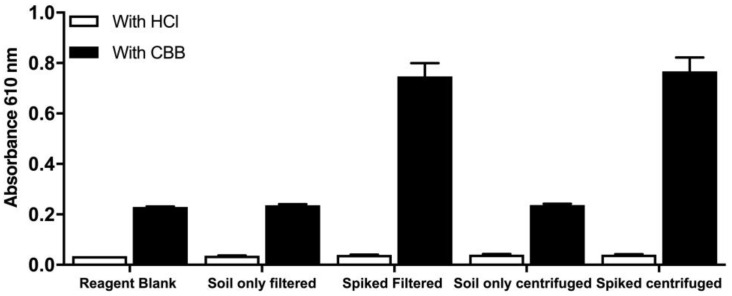
Optimization of the removal of soil debris in the modified protein assay. Spiked BSA (1 μg BSA/mg soil) was recovered in 1 mL ddH_2_O. Controls without CBB (designated as HCl) were included to detect any potential non-protein-absorbing compounds. Data represent average ± SD (N = 3).

To further develop the method, two alternatives for the removal of soil debris were explored. Centrifugation and filtration of the spiked soil extract both proved to be equally effective and reproducible, while blanks without the CBB reagent (designated as HCl) were negligible; thus, we conclude that there are no absorbing compounds that are of non-protein nature (Figure 7).

**Figure 8:**
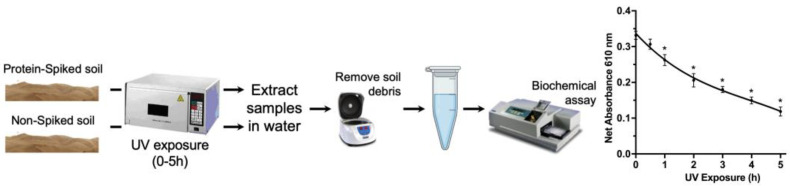
Effect of UV radiation on protein spiked soil. Schematic design, BSA and bacteria protein spiked soil simulants. The line shows the impact of UV exposures modeled with fourth-order polynomial curve fitting. Data represent average ± SD (N = 3) and were considered statistically significant (*) by Student’s *t*-test compared to UV exposure.

To extend our approach to scenarios that would mimic the conditions of an actual soil simulant, the spiked and non-spiked soil simulants were exposed to UV radiation to determine the determine of the impact of UV radiation on proteins. Based on measurements by the Curiosity rover, the total UV radiation dosage at Gale Crater is estimated to be no more than 20 W/m^2^, or 72 kJ/hm^2^. [27]. For the experiment, the same UV dosage of 72 kJ/hm^2^ was utilized for exposure. As a consequence of UV radiation, a decline in the signal of the detected protein was observed in a time-dependent manner, indicating protein degradation and denaturation (Figure 8).

**Figure 9:**
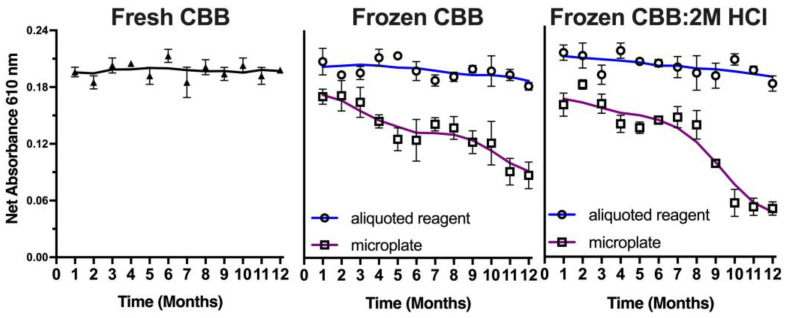
Stability of the CBB reagent over storage time. CBB was stored as aliquots of the CBB reagent frozen mixed with equal volume of 2M HCl frozen, in aliquots (open circles) or frozen on well plates (closed squares). Data represent average ± SD (N = 3).

Since this method meets the criteria for a potential approach as a planetary exploration kit-based approach, the stability of the CBB reagent in storage was explored to evaluate its potential use as a reliable method for detecting proteins as biomarkers of life in extraterrestrial environments. A mission to Mars would require 9 months; hence, the CBB reagent was tested for its stability over time stored at −20 °C (Figure 9). Frozen batches of the reagent were compared to freshly prepared samples, both in pre-plated microwell plates and in bulk aliquots. Samples of 25 μL of CBB reagent, 50 μL of CBB:2M HCl reagent, or aliquots of either of these solutions were periodically thawed and assessed for their sensitivity compared to the freshly prepared CBB reagent. Results revealed a time-dependent decrease in the protein detection signal in the microplate stored dye, with the CBB reagent showing a gradual decrease of 25% after 12 months, while the CBB:2M HCl reagent showed a more significant decrease of 65% after the same storage period. However, the aliquoted reagents remained stable over the 12-month period. These findings suggest that the CBB reagent can be stored in preloaded frozen compartments for future planetary missions, indicating its potential as a reliable method for the detection of proteins as potential biomarkers of life in extraterrestrial environments.

## 4. Discussion

The overall goal of optimizing methods for planetary exploration has been extensively discussed [28]. In order to detect proteins from Martian soil, the ideal approach should need to present high sensitivity and reproducibility, be less (if possible, not at all) prone to contamination and interferences, and be able to survive the adverse conditions and environments until a collected sample can be assayed [29]. Our approach has proven to be promising and fulfills the aforementioned criteria as a simple quantitative approach for the detection of proteins on soil samples. Compared with the traditional Bradford assay, which needs not only Coomassie Brilliant Blue G-250 but also the kosmotropic and protein precipitating reagents [12], the final protocol only requires a filtered CBB reagent diluted with 2M HCl, which can be stable for months.

Although there has been a series of approaches to extract proteins from soils, several caveats have been highlighted. In the study of Chen et al. (2009) [30], a sequential extraction method was designed to remove the main interfering substances. The target soil was extracted sequentially in citrate and SDS buffers and finally extracted by phenol to recover the protein [24]. This method made it possible to obtain the applicable 1-D and 2-D protein profiles as well as find the glomalin-related soil protein. This extraction method was an efficient protocol for soil proteomics; however, this method may not be practical for use in space due to the complexity and the challenges of the space environment, i.e., it will require much effort and face many challenges to achieve the goal as space is a crucial environment. In our method, the extraction of a soil sample is easily performed by vortex, and soil debris can be removed by filtration or centrifugation alternatives. The results showed that using filtration or centrifugation to extract the protein-spiked soil in ddH_2_O, most debris was removed and a clear supernatant was obtained. The absorbance is measured after a small incubation of 10 min and the signal remains stable, which allows the method to be quite effective, fast, and very low-cost.

Another powerful tool that has been proposed for detecting proteins on other planets is the isotope method. This method takes advantage of the isotopic composition of reactants to affect chemical and biochemical reactions, with recent studies demonstrating that isotopic resonance can enhance reaction kinetics [31]. Due to the isotopic composition of elements such as carbon, hydrogen, nitrogen, and oxygen which can vary between living and non-living systems, by analyzing the isotopic composition of a sample, the isotope method could identify such signatures and detect potential life signals. However, the isotope method requires precise control over the isotopic composition of reactants and may not be suitable for use in the harsh and unpredictable conditions of extraterrestrial environments [32]. Additionally, the isotope method may not be effective for detecting proteins specifically, as its focus is on measuring the effects of isotopic composition on reaction kinetics. Hence, in the context of protein detection in extraterrestrial environments such as Mars, the Bradford assay remains a more attractive option due to its ease of use, wide dynamic range, and compatibility with a variety of sample types.

Furthermore, the Signs of Life Detector (SOLID) was designed for identifying and describing organic carbon on Mars by employing cutting-edge liquid extraction and lab-on-a-chip immunoassay techniques [33]. Since 2000, this instrument has undergone significant technological developments. The LDChip biosensor, which serves as the primary element of the SOLID, includes up to 450 antibodies that react to biological materials such as proteins, sugars, or DNA, enabling it to detect the presence of life, be it ancient or modern [34]. This biosensor shares certain similarities with the Bradford assay utilized for protein detection. Both methods rely on the binding of specific molecules to detect the target molecule, which in turn can be indicative of the presence of life. In comparison to LDChip, the Bradford assay is a straightforward and user-friendly method with broad applicability to various types of samples and an extensive dynamic range. Conversely, LDChip necessitates more intricate handling and processing steps, including the introduction of reagents and antibodies, which heightens the likelihood of erroneous results or contamination [35]. Moreover, conducting LDChip analysis requires access to specialized laboratories and skilled personnel for instrument operation and data interpretation, whereas the Bradford assay can be executed in a conventional laboratory environment.

An additional benefit of the implemented Bradford assay was its ability to detect organic proteins. Due to the complex and extreme environment on Mars, such as high levels of radiation and extreme temperature, there were many hypotheses about what life looks like on Mars [36]. One of them was iron-oxidizing bacteria [37]. These bacteria belong to chemolithoautotrophic organisms, which can survive without oxygen and use Fe (II) as an electron donor [26]. Another excellent microorganism that showed the survival capacity to cope with the extreme conditions on Mars was the halophilic archaeon *Hvr* [38]. The study showed that *Hvr* had good resistance to radiation and desiccation under simulated space conditions. In our Bradford assay, the proteins extracted from *E. coli* were detected with a high sensitivity of 0.2 μg protein/mg in spiked soil, which provided a strong indication that the method can be applied for detecting proteins from other microbial sources. While it is acknowledged that *E. coli* is not the same as bacteria and archaea found in Mars-like conditions, it is important to note that *E. coli* is a widely used and well-characterized model organism that has been extensively studied [39]. Furthermore, the use of *E. coli* protein as a positive control is a common practice in microbiological research, as it allows for a standardized and reproducible approach to assessing the performance of a method. Thus, our findings provide a solid foundation for the potential application of the protein extraction method in detecting microbial proteins in extraterrestrial environments.

Considering that UV radiation is a representative factor of the extreme conditions on Mars, it can cause permanent damage to cells at the molecular level leading to cell death [40]. We also considered the impact of UV radiation on proteins that exist on Mars as strong oxidants generated by UV radiation are identified as the leading factor which can cause the degradation of proteins [41]. Due to the UV radiation on Mars being highly variable and dependent on factors such as the location, time of day, and season, we used a dose that was similar to the total UV radiation dosage at Gale Crater which is 72 kJ/hm^2^ [27]. The UV radiation was used in the range of 280–400 nm, which is commonly referred to as UVA and UVB radiation, which is biologically relevant as it causes damage to proteins, DNA, and other biological molecules.

Based on our Bradford assay, protein-spiked soil was exposed to direct UV radiation for various time periods and absorbance was measured. The observed decreasing trend in absorbance with increasing UV radiation exposure time implies that the spiked proteins were degraded by UV radiation. This outcome is in line with the findings of the BIOMEX study. The BIOMEX (Biology and Mars Experiment) experiment aimed to evaluate the impact of space and Mars-like conditions on a range of organisms, including methanogenic archaea, fungi, moss, cyanobacteria, and lichens. Results demonstrated that all organisms exhibited some level of survival, physiological activity, and growth capacity; however, their vital functions declined to varying degrees in simulated or direct space and Mars-like conditions. Methanogenic archaea exhibited greater resistance compared to multicellular life forms, such as fungi and lichens [42].

Despite the promising results of our study, there are several limitations to our assays that need to be acknowledged. Firstly, we did not investigate the effects of perchlorates, cosmic rays, and other potential sources of protein degradation on our protein detection methods. Perchlorates, in particular, have been found in Martian soil and have been shown to have a detrimental effect on the viability of microorganisms. Further studies are needed to determine the potential impact of these factors on our protein detection assays, especially in the context of planetary exploration. Secondly, our assays were developed for Earth-like proteins. It is possible that proteins on Mars, if they exist, may have a different repertoire of amino acids, which could affect their detection using our methods. While the basic chemistry of protein detection is conserved across different amino acid compositions, we cannot rule out the possibility that our assays may not be applicable to Martian proteins. This highlights the need for further investigations into the biochemistry of Martian organisms and the development of assays that can detect a wider range of protein structures. Additionally, it is crucial to consider the necessity of sampling Martian subsurface soils as a possible solution to the challenges of detecting proteins on the surface. Subsurface soils may provide more protection from UV radiation and other environmental factors that can degrade organic compounds, including proteins. Therefore, future studies should explore the feasibility of sampling subsurface soils and developing methods for detecting proteins in these samples to enhance our ability to search for evidence of extraterrestrial life on Mars and other planets [43].

The steps mentioned above can be easily facilitated within an autonomous miniaturized platform, such as the MICRO-life detection platform, which could perform real-time analysis of DNA, RNA, proteins, and other smaller molecules [44] or as part of a bigger mission, especially if the advantage of pre-aliquoted reagent is stored in vessels or well plates ready for use. The field of protein chemistry in soil matrices is under extensive scientific excavation with more and more research articles focusing on proteomic approaches for the identification of proteins in the soil.

The search for evidence of life on Mars has been reinvigorated following the discovery of a potentially habitable environment in the sedimentary record of Gale Crater. In an attempt to detect biosignatures in this environment, a simulated Martian mudstone material was inoculated and cultured before being analyzed using a range of techniques, including bulk elemental analysis-isotope ratio mass spectrometry, laser-ablation ionization mass spectrometry, Raman spectroscopy, and Fourier transform infrared spectroscopy. While high-sensitivity techniques retrieved presumptive biosignatures, the sedimentary matrix presented challenges for all techniques, suggesting that definitive evidence for life may be difficult to detect [45]. Although our approach does not offer an identification outcome (as this is not the reasoning behind it), this method could have significant applications in research related not only to astrobiology but also in the broader field of geosciences and agriculture by providing a low-cost, portable, wide dynamic range, compatible with a variety of sample types, and easy to do method that could be performed directly in the field to assess soil quality in relation to its protein content.

## 5. Conclusions

This study aimed to optimize a method for detecting proteins in Martian soil that is highly sensitive, reproducible, minimally prone to contamination and interferences, and able to withstand the adverse conditions of space environments. The proposed Bradford assay, which requires only a filtered Coomassie Brilliant Blue reagent diluted with hydrochloric acid, proved to be a simple and cost-effective alternative to traditional protein detection methods that require additional reagents. The assay’s ability to detect organic proteins suggests its potential use in confirming the existence of bacteria on Martian soil. The study also investigated the impact of UV radiation on the degradation of proteins in soil and showed that soil layers provide limited protection against direct UV radiation. To overcome the challenges of protein detection on the surface of Mars, sampling subsurface soils may be a possible solution. Subsurface soils may offer better protection from UV radiation and other environmental factors that can degrade organic compounds, including proteins. Thus, future studies should explore the feasibility of sampling subsurface soils and developing methods for detecting proteins in these samples. By addressing this issue, we can improve our ability to search for evidence of extraterrestrial life on Mars and other planets. While the proposed method has potential applications in astrobiology, it requires further validation and optimization to ensure its accuracy and reliability. In conclusion, while our study provides a promising proof of concept for the detection of proteins in Mars-like conditions, there are several limitations that need to be addressed in future studies. Addressing these limitations will be crucial for developing more robust protein detection assays that can support future astrobiological exploration and enhance our understanding of extraterrestrial life.

## Data Availability

Raw data from all biochemical experiments are available on request.
